# Induction of T-cell mitogenic unresponsiveness by recombinant human granulocyte colony-stimulating factor – a reply

**DOI:** 10.1054/bjoc.1999.1185

**Published:** 2000-04-03

**Authors:** E Reyes, E D Bernstein, M Alvarez-Mon

**Affiliations:** Medicine/Immune System Diseases Oncology Service, Department of Medicine, ‘Principe de Asturias' University Hospital, Alcalá University, 28871 Alcalá de Henares, Madrid, Spain


DOI: 10.1054/ bjoc.1999.1185, available online at http://www.idealibrary.com on IDEAL®

### Induction of T-cell mitogenic unresponsiveness by recombinant human granulocyte colony-stimulating factor – a reply

**Sir**,

We are grateful that Rutella et al have brought their impressive research to our attention. We regret that we were not aware of their work prior to the submission of our other recent publication (Reyes E et al, 1999). As they note in their letter to the Editor, combining our investigative results suggests that rHuG-CSF exerts, through the induction of soluble factors such as IL-1 receptor antagonist and TNF soluble receptor, a reversible inhibitor effect on PBMC proliferation that is not due to alterations in cell number ($CD3^+$, $CD19^+$, $CD45^+$, $CD14^+$), cytokine production (IL-1, IL-2, IL-6, IL-10, TNF-α, or IFNγ), or activation status (HLA-DR, CD57, or IL-2 receptor α expression). Rutella et al suggest that the decreased PBMC proliferative response to mitogen after treatment with sera from patients treated with rHuG-CSF is due to cell cycle arrest such that a lymphocyte partial activation phenotype becomes dominant. Interestingly, while failure to progress through $G_0$ is commonly due to alterations in IL-2R expression, we found that rHuG-CSF treatment does not alter the expression of IL-2 receptor alpha (IL-2Rα) on PBMCs. Certainly other avenues leading to inhibition of lymphocyte cycling will have to be explored. Our finding of an upregulation of memory ($CD45RO^+$) T helper cells ($CD4^+$) with rHuG-CSF treatment with a concomittant decline in naive ($CD4^+CD45RA^+$) subset may explain the decreased proliferative response to mitogens, since naive cells demonstrate greater proliferation to mitogenic stimulation than do memory cells. However, this finding cannot explain the lymphocyte cycling arrest in $G_0$. Evaluation of the effects of rHuG-CSF treatment on the expression of co-regulatory molecules on antigen presenting cells may prove fruitful in elucidating the mechanism of lymphocyte cycling arrest.

The ability of rHuG-CSF to inhibit mitogenic proliferative responses may be proven useful clinically in inhibiting unwanted immunologic activity such as acute graft-versus-host disease, autoimmune disorders, as well as chronic inflammatory diseases. Further, Rutella et al's suggestion that rHuG-CSF results in tolerance induction may be exploited by coupling rHuG-CSF treatment with specific antigens in autoimmune disorders. Certainly, more work is needed to establish the mechanism of rHuG-CSF's effect on mitogenic proliferative responses, as well as testing the effect in the context of specific antigens. Still, the previous work of investigators such as Rutella et al, Pan et al, and Roe et al, as well as our own, demonstrates a fruitful role for rHuG-CSF as an immunomodulator in the context of autoimmune/allergy and transplant medicine.

*E Reyes, ED Bernstein, M Alvarez-Mon*
*Medicine/Immune System Diseases Oncology Service,*
*Department of Medicine,*
*'Principe de Asturias' University Hospital,*
*Alcalá University, 28871 Alcalá de Henares, Madrid, Spain*

### REFERENCES

Reyes E, Garcia-Castro I, Esquivel F, Hornedo J, Cortes-Funes H, Solovera J and Alvarez-Mon M (1999) Granulocyte colony-stimulating factor (G-CSF) transiently suppresses mitogen-stimulated T-cell proliferative response. *Br J Cancer* **80**(1/2): 229–235

DOI: 10.1054/ bjoc.1999.1186, available online at http://www.idealibrary.com on IDEAL®

### Methods in molecular biology: minor errors in primer citations with major consequences: how can we minimize these mistakes?

**Sir**,

We read with great interest the article of Forsyth et al (1999) (*Br J Cancer* **79**: 1828–1835) about the role of gene expression of matrix metalloproteinases (MMPs) in malignant gliomas. There is a growing interest in detecting gene expression of these components for a better understanding of molecular mechanisms regarding tumour invasion and metastasis in malignant diseases (Parsons et al, 1997). Therefore, sensitive and specific molecular biological methods are required but they are mostly user orientated and hardly standardized.

We are investigating the molecular biology of prostate cancer, in particular MMPs and their tissue inhibitors, by real-time RT-PCR (Wittwer et al, 1997). Applying the primers for MMP-2 (also named gelatinase A or collagenase IV) used by Forsyth et al for this purpose, we experienced some disagreeable surprises which seem important enough to us to be commented upon.

*© 2000 Cancer Research Campaign* 

